# The involvement of endoplasmic reticulum formation and protein synthesis efficiency in *VCP*- and *ATL1*-related neurological disorders

**DOI:** 10.1186/s12929-017-0403-3

**Published:** 2018-01-08

**Authors:** Yu-Tzu Shih, Yi-Ping Hsueh

**Affiliations:** Institute of Molecular Biology, Academia Sinica, 128, Academia Rd., Sec. 2, Taipei, 11529 Taiwan

**Keywords:** Amyotrophic lateral sclerosis, Atlastin 1, Dendritic spine formation, Endoplasmic reticulum, Frontotemporal dementia, Hereditary spastic paraplegia, Protein synthesis efficiency, Valosin-containing protein

## Abstract

The endoplasmic reticulum (ER) is the biggest organelle in cells and is involved in versatile cellular processes. Formation and maintenance of ER morphology are regulated by a series of proteins controlling membrane fusion and curvature. At least six different ER morphology regulators have been demonstrated to be involved in neurological disorders—including *Valosin-containing protein* (*VCP*), *Atlastin-1* (*ATL1*), *Spastin* (*SPAST*), *Reticulon 2* (*RTN2*), *Receptor expression enhancing protein 1* (*REEP1*) and *RAB10*—suggesting a critical role of ER formation in neuronal activity and function. Among these genes, mutations in *VCP* gene involve in inclusion body myopathy with Paget disease of bone and frontotemporal dementia (IBMPFD), familial amyotrophic lateral sclerosis (ALS), autism spectrum disorders (ASD), and hereditary spastic paraplegia (HSP). *ATL1* is also one of causative genes of HSP. RAB10 is associated with Parkinson’s disease (PD). A recent study showed that VCP and ATL1 work together to regulate dendritic spine formation by controlling ER formation and consequent protein synthesis efficiency. RAB10 shares the same function with VCP and ATL1 to control ER formation and protein synthesis efficiency but acts independently. Increased protein synthesis by adding extra leucine to cultured neurons ameliorated dendritic spine deficits caused by VCP and ATL1 deficiencies, strengthening the significance of protein synthesis in VCP- and ATL1-regulated dendritic spine formation. These findings provide new insight into the roles of ER and protein synthesis in controlling dendritic spine formation and suggest a potential etiology of neurodegenerative disorders caused by mutations in VCP, ATL1 and other genes encoding proteins regulating ER formation and morphogenesis.

## Background

The endoplasmic reticulum (ER) is a contiguous membrane network extending from the nuclear envelope to the entire cytoplasm and making contact with plasma membrane [1–4]. It is responsible for protein synthesis, modification and quality control. The ER also plays crucial roles in carbohydrate metabolism, control of lipid synthesis and delivery, formation of other membrane-bound organelles and lipid droplet and calcium homeostasis [1–3, 5]. The ER undergoes constant extension, retraction and membrane fusion [1, 6–8]. Biogenesis and maintenance of ER are complex and tightly controlled processes [8, 9], and many factors regulating ER formation and morphology have already been identified [1, 7, 8, 10]. Interestingly, mutations in genes involved in the regulation of ER biogenesis and maintenance, such as *Valosin-containing protein* (*VCP*), *Atlastin-1* (*ATL1*), *Spastin* (*SPAST*), *Reticulon 2* (*RTN2*), and *Receptor expression enhancing protein 1* (*REEP1*) have been linked to neurological diseases. *ATL1*, *RTN2*, *SPAST* and *REEP1* are the causative genes of hereditary spastic paraplegia (HSP) [8, 11, 12]. Mutations of the *VCP* (also known as *p97*) gene have been identified in patients with frontotemporal dementia [13, 14], amyotrophic lateral sclerosis (ALS) [15–17], autism spectrum disorders (ASD) [18] and hereditary spastic paraplegia (HSP) [19]. These disease studies highlight the critical role of ER in neuronal function and activity (see Table 1 for a summary). Since ER is critical for many cellular processes, it is important to determine the precise mechanisms of ER involvement in these neurological disorders since such studies are foundation stones in designing potential therapeutics.

**Table 1:** Molecular functions and disease associations of ER morphology regulators

	Disease	Molecular functions
VCP	IBMPFD [13, 14]; ALS [15–17]; ASD [18]; HSP [19]	AAA+ ATPase; molecular chaperon; cofactors guiding different functions [24–26]
ATL1	SPG3A [78]	Dynamin-like GTPase; driving homotypic membrane fusion by dimerization [79].
RTN2	SPG12 [80]	ER shaping protein; interaction with spastin [80].
REEP1	SPG31 [81]	ER-shaping protein; acts together with spastin and atlastin-1 [45].
SPAST	SPG4 [82]	AAA+ ATPase; microtubule-severing protein [83].
RAB10	PD-associated [84]	Small GTPase; controls ER tubule extension and fusion [75]

ER stress is well known to be relevant to neurodegenerative disorders [20–22], making it an excellent downstream candidate of the ER morphology deficits controlling neuronal function. Many excellent reviews have discussed the role of ER stress in neurodegenerative disorders [20–22]. However, a recent study suggests that impairment of protein synthesis efficiency via dysregulation of ER biogenesis and maintenance is critical for dendritic spine deficiencies caused by mutations of three ER morphology regulators, VCP, ATL1 and RAB10 [23]. This finding raises the possibility that, in addition to ER stress, mutations of genetic factors involved in ER formation and the efficiency of downstream protein synthesis may contribute to multiple neurological disorders. In this review, the molecular functions of VCP and ATL1 and their roles in controlling ER formation and protein synthesis efficiency and dendritic spine formation are reviewed and discussed.

## VCP is involved in versatile cellular activities and multiple neurological diseases

VCP, a member of the AAA+ (ATPases Associated with diverse cellular Activities) protein family, acts as a molecular chaperon regulating multiple cellular processes [24–26], including ER-associated protein degradation [27, 28], the ubiquitin–proteasome system [24, 29], ER and Golgi morphogenesis [30–32], chromatin-associated processes, amongst others [24, 33, 34]. These diverse activities are determined by the cofactors of VCP [26]. The two most studied VCP cofactors are the ubiquitin fusion degradation 1-like (UFD1L)-nuclear protein localization homolog 4 (NPL4) heterodimer [35] and P47 [36]. The VCP-UFD1L-NPL4 complex is mainly involved in protein degradation [27, 28, 37] and chromatin-associated processes [24]. When VCP binds to P47, it regulates homotypic membrane fusion of ER and Golgi apparatus [30, 31, 36, 38, 39]. Since VCP uses its N-terminal overlapping binding sites to interact with P47 and the UFD1L-NPL4 dimer [40], expression levels of VCP cofactors may alter complex formation and thereby influence the function of VCP in cells [23].

In 2004, Kimonis and colleagues provided the first evidence that mutations in the *VCP* gene result in inclusion body myopathy with Paget disease of bone and frontotemporal dementia (IBMPFD), which is a multiple tissue disorder associated with myopathy, bony defects and dementia [13]. Later, whole exome sequencing further revealed that VCP is associated with other neurological disorders, including familial ALS [15], ASD [18], and HSP [19]. It is unclear why mutations in a single gene, *VCP*, result in various neurological disorders. Perhaps it is due to the diverse activities of VCP in cells. Since the functions of VCP are determined by its interacting cofactors [26], the genetic diversity and/or expression levels of VCP cofactors likely influence the outcome of VCP deficiency, although direct evidence supporting this hypothesis is lacking.

## ATL1, a causative gene of SPG3A, acts as a membrane fusogen controlling ER formation

Approximately 60% of HSP patients carry autosomal dominant mutations in one of four genes: *ATL1*, *SPAST*, *RTN2* and *REEP1* [10–12, 41]. These four genes work together to drive homotypic ER membrane fusion and coordinate microtubule interactions with the tubular ER network (Table 1) [42–45]. ATL1 acts as a membrane-anchored dynamin-like GTPase and directly interacts with SPAST [46, 47]. The ATL1-SPAST complex also interacts with RTN2 and REEP1 [45, 48, 49]. In addition, *Drosophila* Atlastin functionally associates with TER94 (Transitional endoplasmic reticulum ATPase 94), the VCP ortholog in Drosophila [50]. Mammalian VCP also co-immunoprecipitates with ATL1 [23]—the member of the Atlastin protein family predominantly expressed in the brain [42]—suggesting a physical association of VCP with ATL1 in mammalian brains. Since *VCP* mutation has been identified in patients with HSP [19], it seems plausible that VCP and ATL1 work together to control the function and activity of neurons. We discuss evidence supporting this possibility below.

## Abnormal neuronal morphology as a feature of neurological disorders

Neurons are highly differentiated cells with specialized subcellular structures, including axon, dendrite and synapses. All these subcellular structures are essential for neurons to transmit signals among neurons and required for neuronal function and activity. In mammalian brains, excitatory synapses are mainly localized at the tips of dendritic spines, the tiny protrusions emerging from dendrites [51]. Thus, the morphological features of neurons, such as the size and density of dendritic spines, dendritic arbors and branching level and axonal length, are highly relevant to the function of neurons. The impairments of formation and/or maintenance of these structures result in neuronal defects and neurological disorders. Especially, synaptopathy, such as dendritic spine pathology, is most relevant to many psychiatric, neurodevelopmental and neurodegenerative disorders [52–54]. Morphological change (enlargement, shrinkage or elongation) of dendritic spines and/or alteration (increase or decrease) of dendritic spine density have been demonstrated in various neurological disorders, including Alzheimer’s disease, frontotemporal dementia, schizophrenia, ASD, etc. [52, 55, 56]. The morphological changes of dendritic spines are directly related to synaptic strength and the spine loss reflects a deficit of neuronal connectivity [57–59]. Though electrophysiological studies are still recommended to confirm the conclusion of synaptic deficits, morphological and density analyses of dendritic spines provides the easy and reliable ways to assess synaptic deficits and the potential impairment of neuronal activity. Dendritic spine deficits serve as useful indicator to evaluate pathological condition in various neurological disorders, including neurodevelopmental disorders as well as neurodegenerative diseases.

## *Vcp* deficiency impairs neuronal morphology

Initial evidence supporting a role for VCP in regulating neuronal morphology came from a study about neurofibromin, a protein product encoded by the neurofibromatosis type I (*Nf1*) gene [60–62]. Using a series of biochemical analyses, VCP and P47 were shown to interact with neurofibromin in rat brain extracts and HEK293 cells [60]. Expression of individual VCP- and neurofibromin-interacting domains to disrupt complex formation of neurofibromin and VCP reduced the density of dendritic spines [60]. Furthermore, reduction of *Nf1* and *Vcp* expression decreased dendritic spine density [60]. Thus, the neurofibromin-VCP complex in neurons regulates the formation of excitatory synapses. Since VCP overexpression rescues *Nf1* haploinsufficiency [60] and because the subcellular distribution of VCP is altered in *Nf1*^*+/*−^ mouse brains [60], it would appear that VCP acts downstream of neurofibromin in regulating dendritic spine density.

In addition to dendritic spine formation in mammalian brains, Drosophila *Ter94* is required for dendritic pruning during metamorphosis [63]. *Ter94* deficiency results in mislocalization and gain-of-function of the Drosophila homolog of the human RNA-binding protein TAR**–**DNA-binding protein of 43 k-Daltons. A protein degradation-independent pathway is suggested to be involved in the role of *Ter94* in dendritic pruning [63].

Taken together, the studies in both rodents and Drosophila support the role of VCP in regulation of neuronal morphology. The morphological defects caused by *VCP* deficiency likely impair neuronal function and activity and result in pathological condition. However, the above studies were still limited to in vitro cultured neurons. More in vivo studies using mouse models or patients’ samples are required to verify the results of cultured neurons. It is also intriguing to explore where specific brain region(s) is more susceptible to *NF1* and *VCP* deficiency.

## Involvement of ER morphology and protein synthesis in regulating dendritic spine density

Given the fact that VCP is involved in multiple cellular processes, it has been challenging to investigate the molecular etiology of VCP-related disorders. Since ubiquitin- and VCP-positive protein aggregations in muscle are a hallmark of patients with IBMPFD [13, 64], protein degradation defects caused by *VCP* deficiency have been recognized as an important pathogenic mechanism for VCP-related disorders. However, accumulated evidence suggests that the consequences of VCP deficiency in different types of cells vary. For instance, expression of VCP IBMPFD mutants induces polyubiquitinated protein aggregation in mouse myoblast C2C12 cells [65] but not in cultured hippocampal neurons [60], while still reducing dendritic spine density [23, 60]. These results suggest that another mechanism, in addition to the protein aggregation induced by VCP IBMPFD mutants, is critical for dendritic spine impairment.

Since the functions of VCP are determined by its cofactors, evaluating the roles of VCP’s cofactors in dendritic spine formation may reveal how VCP controls dendritic spine formation. Based on this rationale, two major cofactors of VCP—namely the UFD1L-NPL4 heterodimer and P47—have been knocked down individually in cultured hippocampal neurons. Although the UFD1L-NPL4 heterodimer is well-known to guide VCP’s regulation of protein degradation and chromatin-associated processes [24], knockdown of UFD1L to disrupt the function of the UFD1L-NPL4 heterodimer did not influence the dendritic spine density of cultured hippocampal neurons [23], suggesting that UFD1L-NPL4 heterodimer-dependent processes are not critical to dendritic spine formation. In contrast, knockdown of P47 reduced dendritic spine density [23]. Moreover, P47 overexpression rescued the spine phenotype caused by partially reduced VCP expression using a knockdown approach in cultured neurons, suggesting that P47 acts downstream in VCP-mediated dendritic spine formation [23].

Previous studies indicate a role for the VCP-P47 complex in homotypic membrane fusion of intracellular membrane-bound organelles, particularly ER [32, 36, 66]. Experiments using DsRed-ER (a red fluorescent protein fused with ER-targeting and -retention sequences) to label ER revealed that knockdown of VCP or P47, or overexpression of VCP IBMPFD mutants, indeed reduced the distribution of ER along dendrites in cultured neurons as well as in brains [23]. Further experiments using transmission electron microscopy to analyze knock-in mice carrying the R95G IBMPFD mutation in the *Vcp* gene demonstrated that the length and amounts of rough ER in soma are reduced by VCP IBMPFD mutation [23], supporting that neuronal ER is impaired by *Vcp* deficiency.

In addition to the reduced amounts of rough ER, attachment of ribosomes to rough ER also decreased under expression of VCP IBMPFD mutant [23]. Since ER is critical for the synthesis of membrane, secreted and cytosolic proteins [67–69], a reduction of ribosomal attachment on ER likely has a global effect on the protein synthesis of neurons. The effect of *VCP* deficiency on protein synthesis was directly investigated by bioorthogonal non-canonical amino acid tagging [70] and surface sensing of translation [71]; the former uses L-azidohomoalanine to label newly synthesized proteins, whereas puromycin is integrated into newly synthesized proteins in the latter. Both of these methods revealed that the amount of newly synthesized proteins within 1 h of labeling was reduced under *VCP* deficiency [23]. However, labeled protein amounts after 4 or 6 h were not obviously different between wild type and *VCP*-deficient neurons [23]. This finding indicates that *VCP* deficits impair the efficiency of protein synthesis but not total protein levels, implying that unstable proteins may be more sensitive to *VCP* deficiency.

By increasing protein synthesis to rescue the dendritic spine deficits caused by *VCP* deficiency can further strengthen the notion that inefficient protein synthesis is indeed the key downstream outcome of *VCP* deficiency. The branched-chain amino acid, especially the leucine, is well-known to activate the mTOR pathway that upregulates protein synthesis [72–74]. Adding extra leucine in cultured media increased the protein synthesis of VCP-deficient neurons [23]. Importantly, the dendritic spine defects caused by VCP deficiency were also effectively rescued to levels comparable to those of wild type neurons by leucine supplements [23]. The results of these leucine rescue experiments concluded that VCP mutation or deficiency result in impairment of ER formation and a reduction of protein synthesis efficiency and, consequently, impair dendritic spine formation.

## Convergence of multiple ER formation pathways to control dendritic spine formation

If ER malformation is sufficient to impair protein synthesis efficiency and to result in reduced dendritic spine density, it is reasonable to speculate that other regulators of ER morphology also control protein synthesis efficiency and dendritic spine density. In addition to VCP, many other regulators of ER morphology have been identified. Two other ER morphology regulators, ATL1 and RAB10, have been assessed. RAB10, a small GTPase, regulates ER tubule growth, which is independent of the membrane fusion controlled by ATL1 [75]. Expression of the ATL1 SPG3A mutant or the GDP-locked T23 N mutant of RAB10 impairs ER formation in cultured neurons and reduces protein synthesis efficiency [23]. Importantly, dendritic spine density of cultured hippocampal neurons is reduced by *Atl1* and *Rab10* deficiencies [23]. These studies support the hypothesis that normal ER formation is critical for protein synthesis and for controlling dendritic spine formation.

**Fig. 1:**
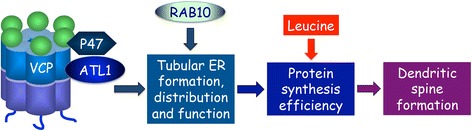
ER formation and consequent protein synthesis efficiency function downstream of multiple factors to control dendritic spine formation. RAB10 and the VCP-P47-ATL1 complex act independently to control tubular ER formation, though both influence protein synthesis efficiency and dendritic spine formation

A previous study suggested that the VCP-P47 complex acts with an unknown membrane fusogen to control homotypic membrane fusion [76]. Since ATL1 functions as an ER fusogen and because ATL1 interacts with VCP [23], ATL1 is therefore an excellent candidate as an interacting partner with VCP to control ER formation and dendritic spine formation. Indeed, in VCP-knockdown neurons, overexpression of wild-type ATL1 increases the density of dendritic spines of cultured hippocampal neurons. Expression of disease-associated mutants of both VCP and ATL1 does not further reduce dendritic spine density compared with single transfected neurons [23]. In contrast, expression of the GDP-locked T23 N mutant of RAB10 further reduces dendritic spine density of neurons expressing the VCP IBMPFD mutant [23]. Taken together, these experiments suggest that ER formation and associated protein synthesis efficiency is a common downstream pathway of multiple upstream regulators (such as VCP-P47-ATL1 and RAB10) controlling dendritic spine formation (Fig. 1).

## Conclusion

Although VCP possesses multiple different functions in cells, its regulation of ER formation is critical for controlling dendritic spine density. Among ER-dependent cellular processes, protein synthesis is particularly important for VCP-, ATL1-, P47- and RAB10-regulated dendritic spine formation. Previous study indicated that tubular rough ER is concentrated at the bases of dendritic spines to meet their demands in response to synaptic stimulation [77]. The studies summarized above provide a mechanism underlying the role of ER and protein synthesis in controlling dendritic spine formation. Nevertheless, several questions remain unanswered. First, whether apart from VCP, ATL1, P47 and RAB10, other regulators of ER morphology have a similar function in protein synthesis and dendritic spine formation. Second, are any specific proteins particularly sensitive to ER malformation? For instance, are short half-life proteins and/or membrane and secreted proteins more susceptible to VCP-, ATL1-, P47- and RAB10-related ER defects? Third, in vivo evidence to support the effect of ER malformation on dendritic spine formation is still lacking. Fourth, since VCP acts downstream of neurofibromin to regulate dendritic spine formation, it would be intriguing to explore whether ER formation and protein synthesis also contribute to neurofibromin-mediated dendritic spine formation. Finally, leucine supplementation seems to be potentially useful for increasing dendritic spine density in vivo. Investigation of the beneficial effects of leucine supplementation on mouse models of VCP- and HSP-related disorders is warranted, potentially providing research avenues for future therapeutics. If protein synthesis efficiency is indeed involved in the etiology of VCP- and HSP-related disorders, it suggests that nutrient and genetic factors may have synergistic effects on induction of these neurodegenerative disorders. Thus, environmental factors, such as nutrients, should also be taken into consideration when investigating VCP- and HSP-related disorders.

## Abbreviations

AAA +: ATPases associated with diverse cellular activities
ALS: amyotrophic lateral sclerosis
ASD: autism spectrum disorders
ATL1: atlastin-1
ER: endoplasmic reticulum
HSP: hereditary spastic paraplegia
IBMPFD: inclusion body myopathy with Paget disease of bone and frontotemporal dementia
NPL4: nuclear protein localization homolog 4
PD: Parkinson’s disease
REEP1: receptor expression enhancing protein 1
RTN2: reticulon 2
SPAST: spastin
SPG: spastic paraplegia
Ter94: transitional endoplasmic reticulum ATPase 94
VCP: valosin-containing protein
UFD1L: ubiquitin fusion degradation 1-like

## References

[1] Chen S, Novick P, Ferro-Novick S (2013). ER structure and function. Curr Opin Cell Biol.

[2] Chang CL, Chen YJ, Liou J (1864). ER-plasma membrane junctions: why and how do we study them?. Biochim Biophys Acta.

[3] Saheki Y, De Camilli P (2017). Endoplasmic reticulum-plasma membrane contact sites. Annu Rev Biochem.

[4] Okeke E, Dingsdale H, Parker T, Voronina S, Tepikin AV (2016). Endoplasmic reticulum-plasma membrane junctions: structure, function and dynamics. J Physiol.

[5] Baumann O, Walz B (2001). Endoplasmic reticulum of animal cells and its organization into structural and functional domains. Int Rev Cytol.

[6] Ching MS, Shen Y, Tan WH, Jeste SS, Morrow EM, Chen X, Mukaddes NM, Yoo SY, Hanson E, Hundley R, Austin C, Becker RE, Berry GT, Driscoll K, Engle EC, Friedman S, Gusella JF, Hisama FM, Irons MB, Lafiosca T, LeClair E, Miller DT, Neessen M, Picker JD, Rappaport L, Rooney CM, Sarco DP, Stoler JM, Walsh CA, Wolff RR, Zhang T, Nasir RH, Wu BL (2010). Deletions of NRXN1 (neurexin-1) predispose to a wide spectrum of developmental disorders. Am J Med Genet B Neuropsychiatr Genet.

[7] McNew JA, Sondermann H, Lee T, Stern M, Brandizzi F (2013). GTP-dependent membrane fusion. Annu Rev Cell Dev Biol.

[8] Goyal U, Blackstone C (1833). Untangling the web: mechanisms underlying ER network formation. Biochim Biophys Acta.

[9] Moss TJ, Daga A, McNew JA (2011). Fusing a lasting relationship between ER tubules. Trends Cell Biol.

[10] Blackstone C, O'Kane CJ, Reid E (2011). Hereditary spastic paraplegias: membrane traffic and the motor pathway. Nat Rev Neurosci.

[11] Renvoise B, Blackstone C (2010). Emerging themes of ER organization in the development and maintenance of axons. Curr Opin Neurobiol.

[12] Fink JK (2013). Hereditary spastic paraplegia: clinico-pathologic features and emerging molecular mechanisms. Acta Neuropathol.

[13] Watts GD, Wymer J, Kovach MJ, Mehta SG, Mumm S, Darvish D, Pestronk A, Whyte MP, Kimonis VE (2004). Inclusion body myopathy associated with Paget disease of bone and frontotemporal dementia is caused by mutant valosin-containing protein. Nat Genet.

[14] Sieben A, Van Langenhove T, Engelborghs S, Martin JJ, Boon P, Cras P, De Deyn PP, Santens P, Van Broeckhoven C, Cruts M (2012). The genetics and neuropathology of frontotemporal lobar degeneration. Acta Neuropathol.

[15] Johnson JO, Mandrioli J, Benatar M, Abramzon Y, Van Deerlin VM, Trojanowski JQ, Gibbs JR, Brunetti M, Gronka S, Wuu J, Ding J, McCluskey L, Martinez-Lage M, Falcone D, Hernandez DG, Arepalli S, Chong S, Schymick JC, Rothstein J, Landi F, Wang YD, Calvo A, Mora G, Sabatelli M, Monsurro MR, Battistini S, Salvi F, Spataro R, Sola P, Borghero G, Galassi G, Scholz SW, Taylor JP, Restagno G, Chio A, Traynor BJ (2010). Exome sequencing reveals VCP mutations as a cause of familial ALS. Neuron.

[16] Koppers M, van Blitterswijk MM, Vlam L, Rowicka PA, van Vught PW, Groen EJ, Spliet WG, Engelen-Lee J, Schelhaas HJ, de Visser M, van der Kooi AJ, van der Pol WL, Pasterkamp RJ, Veldink JH, van den Berg LH (2012). VCP mutations in familial and sporadic amyotrophic lateral sclerosis. Neurobiol Aging.

[17] Gonzalez-Perez P, Woehlbier U, Chian RJ, Sapp P, Rouleau GA, Leblond CS, Daoud H, Dion PA, Landers JE, Hetz C, Brown RH (2015). Identification of rare protein disulfide isomerase gene variants in amyotrophic lateral sclerosis patients. Gene.

[18] Iossifov I, Ronemus M, Levy D, Wang Z, Hakker I, Rosenbaum J, Yamrom B, Lee YH, Narzisi G, Leotta A, Kendall J, Grabowska E, Ma B, Marks S, Rodgers L, Stepansky A, Troge J, Andrews P, Bekritsky M, Pradhan K, Ghiban E, Kramer M, Parla J, Demeter R, Fulton LL, Fulton RS, Magrini VJ, Ye K, Darnell JC, Darnell RB, Mardis ER, Wilson RK, Schatz MC, McCombie WR, Wigler M (2012). De novo gene disruptions in children on the autistic spectrum. Neuron.

[19] van de Warrenburg BP, Schouten MI, de Bot ST, Vermeer S, Meijer R, Pennings M, Gilissen C, Willemsen MA, Scheffer H, Kamsteeg EJ (2016). Clinical exome sequencing for cerebellar ataxia and spastic paraplegia uncovers novel gene-disease associations and unanticipated rare disorders. Eur J Hum Genet.

[20] Manifava M, Smith M, Rotondo S, Walker S, Niewczas I, Zoncu R, Clark J, Ktistakis NT. Dynamics of mTORC1 activation in response to amino acids. elife. 2016;5:e19960. 10.7554/eLife.19960 PMC5059141 27725083

[21] Martinez G, Duran-Aniotz C, Cabral-Miranda F, Vivar JP, Hetz C (2017). Endoplasmic reticulum proteostasis impairment in aging. Aging Cell.

[22] Remondelli P, Renna M (2017). The endoplasmic reticulum unfolded protein response in neurodegenerative disorders and its potential therapeutic significance. Front Mol Neurosci.

[23] Shih YT, Hsueh YP (2016). VCP and ATL1 regulate endoplasmic reticulum and protein synthesis for dendritic spine formation. Nat Commun.

[24] Meyer H, Bug M, Bremer S (2012). Emerging functions of the VCP/p97 AAA-ATPase in the ubiquitin system. Nat Cell Biol.

[25] Meyer H, Weihl CC (2014). The VCP/p97 system at a glance: connecting cellular function to disease pathogenesis. J Cell Sci.

[26] Buchberger A, Schindelin H, Hanzelmann P (2015). Control of p97 function by cofactor binding. FEBS Lett.

[27] Ye Y, Meyer HH, Rapoport TA (2001). The AAA ATPase Cdc48/p97 and its partners transport proteins from the ER into the cytosol. Nature.

[28] Jarosch E, Taxis C, Volkwein C, Bordallo J, Finley D, Wolf DH, Sommer T (2002). Protein dislocation from the ER requires polyubiquitination and the AAA-ATPase Cdc48. Nat Cell Biol.

[29] Dai RM, Chen E, Longo DL, Gorbea CM, Li CC (1998). Involvement of valosin-containing protein, an ATPase co-purified with IkappaBalpha and 26 S proteasome, in ubiquitin-proteasome-mediated degradation of IkappaBalpha. J Biol Chem.

[30] Latterich M, Frohlich KU, Schekman R (1995). Membrane fusion and the cell cycle: Cdc48p participates in the fusion of ER membranes. Cell.

[31] Lavoie C, Chevet E, Roy L, Tonks NK, Fazel A, Posner BI, Paiement J, Bergeron JJ (2000). Tyrosine phosphorylation of p97 regulates transitional endoplasmic reticulum assembly in vitro. Proc Natl Acad Sci U S A.

[32] Vedrenne C, Hauri HP (2006). Morphogenesis of the endoplasmic reticulum: beyond active membrane expansion. Traffic.

[33] Ju JS, Weihl CC (2010). P97/VCP at the intersection of the autophagy and the ubiquitin proteasome system. Autophagy.

[34] Tresse E, Salomons FA, Vesa J, Bott LC, Kimonis V, Yao TP, Dantuma NP, Taylor JP (2010). VCP/p97 is essential for maturation of ubiquitin-containing autophagosomes and this function is impaired by mutations that cause IBMPFD. Autophagy.

[35] Meyer HH, Wang Y, Warren G (2002). Direct binding of ubiquitin conjugates by the mammalian p97 adaptor complexes, p47 and Ufd1-Npl4. EMBO J.

[36] Kondo H, Rabouille C, Newman R, Levine TP, Pappin D, Freemont P, Warren G (1997). p47 is a cofactor for p97-mediated membrane fusion. Nature.

[37] Meyer HH, Shorter JG, Seemann J, Pappin D, Warren G (2000). A complex of mammalian ufd1 and npl4 links the AAA-ATPase, p97, to ubiquitin and nuclear transport pathways. EMBO J.

[38] Kano F, Kondo H, Yamamoto A, Tanaka AR, Hosokawa N, Nagata K, Murata M (2005). The maintenance of the endoplasmic reticulum network is regulated by p47, a cofactor of p97, through phosphorylation by cdc2 kinase. Genes Cells.

[39] Roy L, Bergeron JJ, Lavoie C, Hendriks R, Gushue J, Fazel A, Pelletier A, Morre DJ, Subramaniam VN, Hong W, Paiement J (2000). Role of p97 and syntaxin 5 in the assembly of transitional endoplasmic reticulum. Mol Biol Cell.

[40] Bruderer RM, Brasseur C, Meyer HH (2004). The AAA ATPase p97/VCP interacts with its alternative co-factors, Ufd1-Npl4 and p47, through a common bipartite binding mechanism. J Biol Chem.

[41] Guelly C, Zhu PP, Leonardis L, Papic L, Zidar J, Schabhuttl M, Strohmaier H, Weis J, Strom TM, Baets J, Willems J, De Jonghe P, Reilly MM, Frohlich E, Hatz M, Trajanoski S, Pieber TR, Janecke AR, Blackstone C, Auer-Grumbach M (2011). Targeted high-throughput sequencing identifies mutations in atlastin-1 as a cause of hereditary sensory neuropathy type I. Am J Hum Genet.

[42] Rismanchi N, Soderblom C, Stadler J, Zhu PP, Blackstone C (2008). Atlastin GTPases are required for Golgi apparatus and ER morphogenesis. Hum Mol Genet.

[43] Hu J, Shibata Y, Zhu PP, Voss C, Rismanchi N, Prinz WA, Rapoport TA, Blackstone C (2009). A class of dynamin-like GTPases involved in the generation of the tubular ER network. Cell.

[44] Orso G, Pendin D, Liu S, Tosetto J, Moss TJ, Faust JE, Micaroni M, Egorova A, Martinuzzi A, McNew JA, Daga A (2009). Homotypic fusion of ER membranes requires the dynamin-like GTPase atlastin. Nature.

[45] Park SH, Zhu PP, Parker RL, Blackstone C (2010). Hereditary spastic paraplegia proteins REEP1, spastin, and atlastin-1 coordinate microtubule interactions with the tubular ER network. J Clin Invest.

[46] Sanderson CM, Connell JW, Edwards TL, Bright NA, Duley S, Thompson A, Luzio JP, Reid E (2006). Spastin and atlastin, two proteins mutated in autosomal-dominant hereditary spastic paraplegia, are binding partners. Hum Mol Genet.

[47] Evans K, Keller C, Pavur K, Glasgow K, Conn B, Lauring B (2006). Interaction of two hereditary spastic paraplegia gene products, spastin and atlastin, suggests a common pathway for axonal maintenance. Proc Natl Acad Sci U S A.

[48] Connell JW, Lindon C, Luzio JP, Reid E (2009). Spastin couples microtubule severing to membrane traffic in completion of cytokinesis and secretion. Traffic.

[49] Mannan AU, Boehm J, Sauter SM, Rauber A, Byrne PC, Neesen J, Engel W (2006). Spastin, the most commonly mutated protein in hereditary spastic paraplegia interacts with Reticulon 1 an endoplasmic reticulum protein. Neurogenetics.

[50] O'Sullivan NC, Drager N, O'Kane CJ (2013). Characterization of the drosophila Atlastin Interactome reveals VCP as a functionally related Interactor. J Genet Genomics.

[51] Harris KM, Stevens JK (1989). Dendritic spines of CA 1 pyramidal cells in the rat hippocampus: serial electron microscopy with reference to their biophysical characteristics. J Neurosci.

[52] Herms J, Dorostkar MM (2016). Dendritic spine pathology in neurodegenerative diseases. Annu Rev Pathol.

[53] Roeper J (2017). Closing gaps in brain disease-from overlapping genetic architecture to common motifs of synapse dysfunction. Curr Opin Neurobiol.

[54] Reig-Viader R, Sindreu C, Bayes A. Synaptic proteomics as a means to identify the molecular basis of mental illness: are we getting there? Prog Neuro-Psychopharmacol Biol Psychiatry. 2017; 10.1016/j.pnpbp.2017.09.011 28941771

[55] Penzes P, Cahill ME, Jones KA, VanLeeuwen JE, Woolfrey KM (2011). Dendritic spine pathology in neuropsychiatric disorders. Nat Neurosci.

[56] Kulkarni VA, Firestein BL (2012). The dendritic tree and brain disorders. Mol Cell Neurosci.

[57] Tada T, Sheng M (2006). Molecular mechanisms of dendritic spine morphogenesis. Curr Opin Neurobiol.

[58] Lee SH, Sheng M (2000). Development of neuron-neuron synapses. Curr Opin Neurobiol.

[59] Hering H, Sheng M (2001). Dendritic spines: structure, dynamics and regulation. Nat Rev Neurosci.

[60] Wang HF, Shih YT, Chen CY, Chao HW, Lee MJ, Hsueh YP (2011). Valosin-containing protein and neurofibromin interact to regulate dendritic spine density. J Clin Invest.

[61] Hsueh YP (2012). From neurodevelopment to neurodegeneration: the interaction of neurofibromin and valosin-containing protein/p97 in regulation of dendritic spine formation. J Biomed Sci.

[62] Hsueh YP (2007). Neurofibromin signaling and synapses. J Biomed Sci.

[63] Rumpf S, Bagley JA, Thompson-Peer KL, Zhu S, Gorczyca D, Beckstead RB, Jan LY, Jan YN (2014). Drosophila Valosin-containing protein is required for dendrite pruning through a regulatory role in mRNA metabolism. Proc Natl Acad Sci U S A.

[64] Schroder R, Watts GD, Mehta SG, Evert BO, Broich P, Fliessbach K, Pauls K, Hans VH, Kimonis V, Thal DR (2005). Mutant valosin-containing protein causes a novel type of frontotemporal dementia. Ann Neurol.

[65] Weihl CC, Dalal S, Pestronk A, Hanson PI (2006). Inclusion body myopathy-associated mutations in p97/VCP impair endoplasmic reticulum-associated degradation. Hum Mol Genet.

[66] Uchiyama K, Kondo H (2005). p97/p47-mediated biogenesis of Golgi and ER. J Biochem.

[67] Reid DW, Nicchitta CV (2015). Diversity and selectivity in mRNA translation on the endoplasmic reticulum. Nat Rev Mol Cell Biol.

[68] Potter MD, Nicchitta CV (2000). Regulation of ribosome detachment from the mammalian endoplasmic reticulum membrane. J Biol Chem.

[69] Stephens SB, Nicchitta CV (2008). Divergent regulation of protein synthesis in the cytosol and endoplasmic reticulum compartments of mammalian cells. Mol Biol Cell.

[70] Dieterich DC, Link AJ, Graumann J, Tirrell DA, Schuman EM (2006). Selective identification of newly synthesized proteins in mammalian cells using bioorthogonal noncanonical amino acid tagging (BONCAT). Proc Natl Acad Sci U S A.

[71] Schmidt EK, Clavarino G, Ceppi M, Pierre P (2009). SUnSET, a nonradioactive method to monitor protein synthesis. Nat Methods.

[72] Jewell JL, Russell RC, Guan KL (2013). Amino acid signalling upstream of mTOR. Nat Rev Mol Cell Biol.

[73] Jewell JL, Kim YC, Russell RC, Yu FX, Park HW, Plouffe SW, Tagliabracci VS, Guan KL (2015). Metabolism. Differential regulation of mTORC1 by leucine and glutamine. Science.

[74] Wolfson RL, Chantranupong L, Saxton RA, Shen K, Scaria SM, Cantor JR, Sabatini DM (2016). Sestrin2 is a leucine sensor for the mTORC1 pathway. Science.

[75] English AR, Voeltz GK (2013). Rab10 GTPase regulates ER dynamics and morphology. Nat Cell Biol.

[76] Meyer HH (2005). Golgi reassembly after mitosis: the AAA family meets the ubiquitin family. Biochim Biophys Acta.

[77] Cui-Wang T, Hanus C, Cui T, Helton T, Bourne J, Watson D, Harris KM, Ehlers MD (2012). Local zones of endoplasmic reticulum complexity confine cargo in neuronal dendrites. Cell.

[78] Zhang W, Rhodes SD, Zhao L, He Y, Zhang Y, Shen Y, Yang D, Wu X, Li X, Yang X, Park SJ, Chen S, Turner C, Yang FC (2011). Primary osteopathy of vertebrae in a neurofibromatosis type 1 murine model. Bone.

[79] Daumke O, Praefcke GJ (2011). Structural insights into membrane fusion at the endoplasmic reticulum. Proc Natl Acad Sci U S A.

[80] Montenegro G, Rebelo AP, Connell J, Allison R, Babalini C, D'Aloia M, Montieri P, Schule R, Ishiura H, Price J, Strickland A, Gonzalez MA, Baumbach-Reardon L, Deconinck T, Huang J, Bernardi G, Vance JM, Rogers MT, Tsuji S, De Jonghe P, Pericak-Vance MA, Schols L, Orlacchio A, Reid E, Zuchner S (2012). Mutations in the ER-shaping protein reticulon 2 cause the axon-degenerative disorder hereditary spastic paraplegia type 12. J Clin Invest.

[81] Zuchner S, Wang G, Tran-Viet KN, Nance MA, Gaskell PC, Vance JM, Ashley-Koch AE, Pericak-Vance MA (2006). Mutations in the novel mitochondrial protein REEP1 cause hereditary spastic paraplegia type 31. Am J Hum Genet.

[82] Sauter S, Miterski B, Klimpe S, Bonsch D, Schols L, Visbeck A, Papke T, Hopf HC, Engel W, Deufel T, Epplen JT, Neesen J (2002). Mutation analysis of the spastin gene (SPG4) in patients in Germany with autosomal dominant hereditary spastic paraplegia. Hum Mutat.

[83] Errico A, Ballabio A, Rugarli EI (2002). Spastin, the protein mutated in autosomal dominant hereditary spastic paraplegia, is involved in microtubule dynamics. Hum Mol Genet.

[84] Steger M, Tonelli F, Ito G, Davies P, Trost M, Vetter M, Wachter S, Lorentzen E, Duddy G, Wilson S, Baptista MA, Fiske BK, Fell MJ, Morrow JA, Reith AD, Alessi DR, Mann M. Phosphoproteomics reveals that Parkinson's disease kinase LRRK2 regulates a subset of Rab GTPases. elife. 2016;5:e12813. 10.7554/eLife.12813 PMC4769169 26824392

